# Body Image Dissatisfaction and Impulse Buying: A Moderated Mediation Model

**DOI:** 10.3389/fpsyg.2021.653559

**Published:** 2021-04-26

**Authors:** Zhihui Cai, Yang Gui, Dandan Wang, Han Yang, Peipei Mao, Zhikeng Wang

**Affiliations:** ^1^School of Psychology, Central China Normal University, Wuhan, China; ^2^Key Laboratory of Adolescent Cyberpsychology and Behavior, Ministry of Education, Wuhan, China

**Keywords:** body image dissatisfaction, impulse buying, self-acceptance, self-esteem, moderated mediation

## Abstract

This study investigated the mediating roles of self-acceptance and self-esteem in the relationship between body image dissatisfaction and impulse buying, and tested the moderating effect of gender on the relationships. A sample of 374 college students and graduate students (33.4% male, 66.6% female) participated in the study. Results revealed that (a) body image dissatisfaction positively associated with impulse buying; (b) self-esteem plays a mediating role between body image dissatisfaction and impulsive buying; (c) a serial indirect pathway (i.e., Body image dissatisfaction → self-acceptance → self-esteem → impulse buying) emerged; (d) the mediation path from self-acceptance to self-esteem was stronger for female than that from male. The results underscore the importance of identifying the mechanisms that moderate the mediated path between body image dissatisfaction and impulse buying among students. These findings point to the potential implications about how to reduce impulse buying through improving body image satisfaction.

## Introduction

Impulse buying is defined as a spontaneous and unplanned purchase, which is characterized by quick purchase decisions, with little or no evaluation of consequences (Rook, 1987). With the rapid development of social economy, the proportion of impulse buying in our daily purchasing activities is increasing and impulse buying has gradually become a mainstream phenomenon in China (Lu et al., 2015). Impulse buying has its positive side, such as alleviating people's negative emotions and promoting self-image (Lucas and Koff, 2017). However, impulsive buying could lead to negative consequences. It could result in negative emotions such as guilt (Li et al., 2015) and lower levels of subjective well-being (Silvera et al., 2008), which could further affect mental health (Rook, 1987); it causes young people to fall into excessive consumption (Forney and Park, 2009), which leads to serious debt problems and even suicide behavior (Yi and Baumgartner, 2011; R3, 2018). Therefore, the formation mechanism of impulsive buying has been deeply explored, which is of great significance for advocating correct and rational consumer behavior.

Body self is an important part of the self-concept. According to the psychodynamics point of view, the body self includes the individual's body image (Huang et al., 2002). Body image is a subjective, comprehensive, and evaluative concept of an individual's physical characteristics, which includes the individual's perception and evaluation of their characteristics and what the individual feels comes from the evaluation of his body image by others (Gallagher, 2005). Previous studies have found that perceptions, feelings, and attitudes to the body and appearance have an important impact on impulse buying (Lucas and Koff, 2017). However, empirical research on the relationship between body image and impulsive buying and research on the internal mechanism is relatively rare. Therefore, this research focuses on the variable of body image dissatisfaction, and explores its mechanism of action with impulse buying.

Body image dissatisfaction refers to people's negative evaluation of their body image, which may have a negative impact on emotional health such as producing negative emotions (Lucas and Koff, 2017) and depression (Fung et al., 2010). Self-regulation theory states that people are driven by two different types of motivations to change their behaviors, thoughts, and emotions to meet their own internal standards. One is the motivation to gain positive results, and the other is the preventive motivation to avoid negative results (Higgins, 2002). Verplanken and Sato (2011) believe that impulse buying may have a self-regulation function, such as providing buyers with required identity symbols (improving motivation) or improving low self-esteem (preventing motivation). A large number of studies have shown that appearance enhancing motive is the core element of impulse buying (Durante et al., 2010). In order to maintain and enhance self-attraction and self-image, people are more likely to purchase related products, such as fashionable clothes, beauty products, etc. (Dittmar et al., 1995). Besides, studies have found that unsatisfactory body image could cause individuals to produce negative emotions. People will conduct impulse buying in the cause of alleviating the pain caused by negative evaluation of body image (Lucas and Koff, 2017). Therefore, we assumed that body image dissatisfaction had a positive predictive effect on impulse buying (H1).

So, how does body image dissatisfaction lead to impulse buying? Previous studies have explored from the perspective of negative emotions (Lucas and Koff, 2017). However, there is still a lack of research on the mechanism of personal self-acceptance and self-esteem in the relationship between body image dissatisfaction and impulse buying. Therefore, current research introduces self-acceptance and self- esteem, constructs an integrated mediation model, and explores the integrated mediation effect of self-acceptance and self-esteem in the relationship between body image dissatisfaction and impulse buying.

Self-acceptance refers to people taking a positive attitude toward themselves and all their characteristics (Sun and Lu, 2017). Self-acceptance reflects the degree of individual acceptance of oneself. Higher levels of self-acceptance indicate higher levels of self-identification, which may reduce the tendency to use goods as self-projections. Ryff and Singer (1996) psychological well-being theory believes that self-acceptance is an important component and indicator of individual psychological well-being, and self-acceptors tend to have stronger happiness experiences and more positive emotions. At the same time, some studies have shown that self-acceptance is significantly positively correlated with positive emotions (Jimenez et al., 2010). Emotions are an important factor affecting impulse buying. Studies have shown that positive emotions could enhance consumers' buying drive more, thus promoting them to make impulse purchases (Fan and Zhang, 2006; Lu et al., 2015). Therefore, self- acceptance may affect impulse buying. In addition, self-acceptance is also affected by dissatisfaction with body image. Previous studies have found that body image and self-acceptance are positively correlated (Mcguire et al., 2016; Wallwiener et al., 2016; Xie et al., 2018; Wang, 2019). They believe that people with positive body images have correct self-knowledge and thus accept themselves. The body is part of the self. People who have negative self-evaluations of their bodies tend to have negative self-evaluations, which leads to lower levels of self-acceptance. Therefore, individuals with body image dissatisfaction may have a lower degree of self-acceptance. Based on that, our study hypothesized that self-acceptance would mediate the association between dissatisfaction with body image and impulse buying (H2).

As a trait, self-esteem is an important part of self-concept and refers to an individual's overall view of himself as valuable or worthless (Baumeister, 1999). Low self-esteem is the result of individuals comparing themselves to a self-determined standard of value. If there is any negative inconsistency between a person's “self” and his or her perceived value, it could lead to a feeling of low self-esteem. Some researchers believe that body image is positively correlated with self-esteem, and body dissatisfaction could significantly predict low self-esteem. In other words, the lower self-esteem of people dissatisfied with their own body (Littleton and Ollenclick, 2003; Johnson and Wardle, 2005; Paxton et al., 2006; Ping et al., 2011). In addition, a person with low self-esteem will have a feeling of inadequate self, which makes him/her feel stressed and emotionally vulnerable (Higgins, 1987). In order to relieve psychological pressure and bridge the gap, individuals may engage in impulse buying. The theory of “compensatory consumption” (Pettit and Sivanathan, 2011) believes that people with low self-esteem tend to restore their self-esteem by compensating for material goods. Some studies also found that self-esteem could negatively predict impulse buying (Silvera et al., 2008; Bandyopadhyay, 2016). Therefore, this study assumes that unsatisfactory body image could further affect impulse buying through the mediation of self-esteem (H3).

Furthermore, self-esteem is also affected by self-acceptance. In his 1998 book “The Six Brands of Self-Esteem,” Nathaniel Branden, the founder of the psychology of self-esteem, argues that self-acceptance is the second pillar of self-esteem and the basis on which self-esteem grows (Nathaniel, 1998). He pointed out that self-acceptance, as the driving force of self-esteem, could promote the healthy development of self-esteem. Hence self-acceptance could positively predict the level of individual self-esteem. Relevant studies have also confirmed this point (Gao and Cong, 2000; Zhang, 2016; Zhu, 2016). Therefore, this study further hypothesized that body image dissatisfaction was positively related to impulse buying through the integrated mediation of self- acceptance and self- esteem (H4).

According to the theory of evolution, men pay more attention to female reproductive ability when choosing a spouse, so they pay more attention to the appearance and figure that could reflect female reproductive ability; While women pay more attention to the ability of men to nurture offspring when choosing a spouse, so women are more likely to choose a capable, ambitious and accomplished man (Wang, 2012; Wei, 2016). That is to say, there could be gender differences in the dimension of self among individuals. Males pay more attention to their inner self, while females pay more attention to their outer self. Self-acceptance means that people have a positive attitude toward their inner and outer selves. Due to the differences in self-care between males and females, there may be gender differences in individual self-acceptance. Previous studies have also shown that males and females show different self-acceptance (Li, 2003). According to the important weighted model of self-esteem, the degree to which an individual attaches importance to a certain dimension of self will affect the overall level of self-esteem. The more an individual values a particular dimension of the self, the greater its impact on overall self-esteem (Pelham and Swann, 1989; Pelham, 1995; Tajfel, 2003). This also suggests that there could be gender differences in the relationship between self-acceptance and self-esteem. Previous studies have also found that a man's qualities predict his self-esteem more than looks do. For women, only appearance significantly predicted their self-esteem (Peng et al., 2018). Accordingly, this study hypothesized that gender had a moderating effect on the effect of self-acceptance on self-esteem (H5).

**Figure 1:**
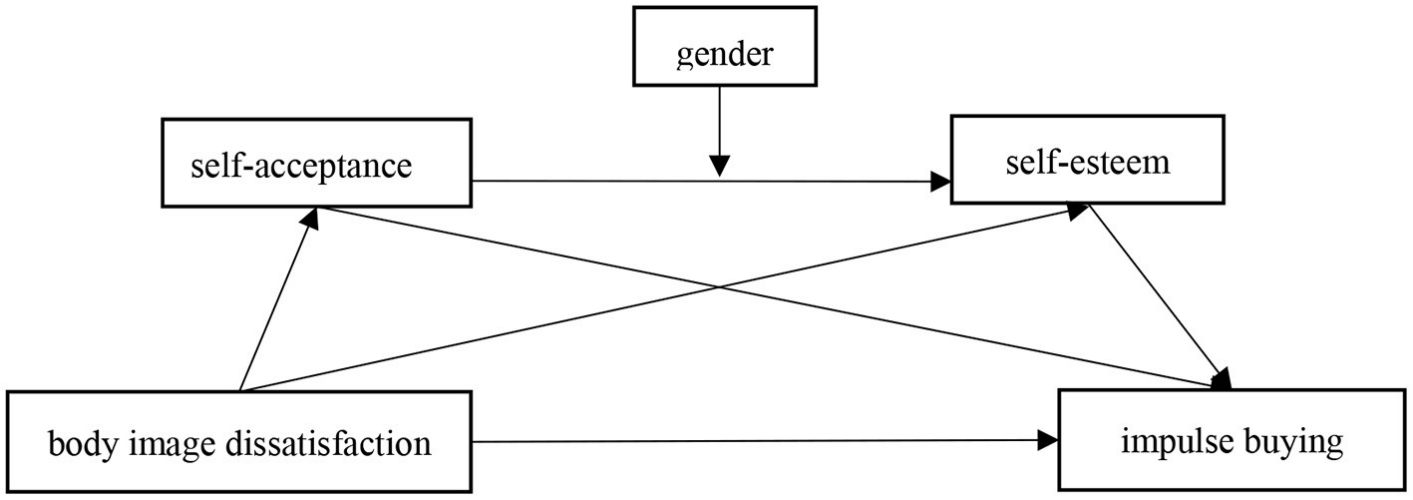
Conceptual model.

To sum up, based on the self-regulation theory, compensatory consumption theory, the perspective of body intention and the results of relevant empirical studies, this study intends to construct an integrated moderated mediation model (As shown in Figure 1). In a nutshell, self-acceptance and self-esteem mediate the relationship between the body image dissatisfaction and impulsive buying. And gender, as a moderator variable, moderates the path of self-acceptance into self-esteem. Specifically, for men, internal self-acceptance is a better predictor of self-esteem than external self-acceptance. For women, external acceptance was a stronger predictor of self-esteem than internal self-acceptance.

## Methods

### Participants

The protocol of the current study was approved by the institutional review boards of the university with which the authors were affiliated with. The participants were undergraduate and graduate students recruited from two universities in the province of Hubei. Pencil-and-paper questionnaires and also the online survey were administered to the participants. Finally, a total of 374 college students and graduate students aged from 16 to 31 years old (*M* = 20.68, *SD* = 2.31) participated in present survey. Of the participants, 59 (15.8%) were freshmen, 136 (36.4%) were sophomores, 40 (10.7%) were juniors, 48 (12.8%) were seniors, 85 (22.7%) were post-graduate students, and 6 (1.6%) were doctoral candidates; 149 (39.8%) were liberal arts majors, 178 (47.6%) were science majors, 24 (6.4%) were engineering majors, and 23(6.1%) were art majors; 125 (33.4%) were male, 249 (66.6%) were female.

### Procedures

Data were collected in university in September 2019. Graduate students of psychology department were trained to gather the data and administered the questionnaires using standardized procedures to ensure the standardization of the data collection process. Informed consent was obtained from all of the participants before data collection. Questionnaires were distributed centrally in classroom. After all the participants answered, the questionnaires were collected.

### Measures

#### Body Image Dissatisfaction

The Simplified Negative Body Image Scale was used to measure body image dissatisfaction, which was compiled by Chen et al. (2006) and revised by Liu (2009). The scale divided negative body image into one general factor: overall body dissatisfaction and four main special factors: fat, thin, appearance, and short. The scale included 27 items (e.g., “I am worried about my appearance”) to which Participants responded on a 5-point Likert-type scale with values ranging from 1 (strongly disagree) to 5 (strongly agree). Higher scores indicated a greater level of body image dissatisfaction. The Cronbach's α of the scale in this study was 0.877.

#### Self-Acceptance

We used the Self-acceptance Questionnaire (Cong and Gao, 1999) to assess the degree of self-acceptance. The questionnaire is divided into two dimensions: self-acceptance and self-evaluation. Participants responded to items on a 4-point Likert scale with values ranging from 1 (strongly disagree) to 4 (strongly agree) on the 16 items in the questionnaire. A typical item was “I'm almost all strengths and specialties.” The higher the score, the higher the level of self-acceptance. In our study, the Cronbach's α of the scale was 0.844.

#### Self-Esteem

The 10-item Chinese revision of the Rosenberg Self-Esteem Scale (Shen and Cai, 2008) was used to measure self-esteem. Each item was rated on a scale from 1 (strongly agree) to 4 (strongly disagree). Higher scores indicated higher self-esteem. A typical item was “I can do as well as most people.” In this study, Cronbach's α for self- esteem was 0.878.

#### Impulse Buying

We measured impulse buying by using the Chinese Consumers' Impulse Buying Tendency Scale (Jing and Yue, 2005), which included 27 items. Two dimensions was contained in this scale, one focusing on cognitive aspects (e.g., “I have a plan to buy things.”), and the other focusing on affective aspects (e.g., “I will buy something to change my mood.”). Students responded on a 7-point Likert scale with values ranging from 1 (strongly disagree) to 7 (strongly agree). Higher scores indicated that people have a stronger impulse buying tendency. In our study, Cronbach's α for impulse buying was 0.905.

### Statistical Analysis

All statistical analyses were conducted by using SPSS 26.0. We calculated the descriptive statistics for all of variables. Pearson correlations were calculated to test the bivariate associations among body image dissatisfaction, impulse buying, self-acceptance and self-esteem. We tested the moderated mediation model using model 91 of the PROCESS macro (http://www.afhayes.com, Hayes, 2013). This approach has been developed and widely used for testing complex models that include both mediating and moderating variables (e.g., Liu et al., 2019; Zheng et al., 2020). The bootstrapping method was used to test the mediation effects. This method produced 95% bias-corrected confidence intervals of the estimates from 5,000 re-samples of the data. Confidence interval that did not contain zero indicate a significant effect. As previous studies have found that students' age was associated with impulse buying (Coley and Burgess, 2003; Vohs and Faber, 2007), we controlled students' age in our analyses.

## Results

### Preliminary Analyses

The descriptive statistics and correlation matrix for all the variables were presented in Table 1. The missing data were processed by Full Information Maximizing-Likelihood (FIML). Body image dissatisfaction was negatively correlated with self-acceptance and self-esteem and positively correlated with impulse buying. Self-acceptance was positively correlated with self-esteem and negatively correlated with impulse buying. Self-esteem was negatively correlated with impulse buying. The relationship between the variables supports subsequent model testing.

**Table 1:** Descriptive statistics and correlations between variables.

**Variables**	***M***	***SD***	**1**	**2**	**3**	**4**	**5**	**6**
1. Gender	0.33	0.47	-					
2. Age	20.68	2.31	0.02	-				
3. Body image dissatisfaction	2.58	0.57	0.02	−0.10	-			
4. Self-acceptance	2.45	0.42	−0.03	0.13^*^	−0.43^***^	-		
5. Self-esteem	2.85	0.51	0.00	−0.03	−0.50^***^	0.71^***^	-	
6. Impulse buying	3.37	0.82	0.06	0.06	0.35^***^	−0.19^***^	−0.33^***^	-

*Gender was dummy coded such that 0 = male and 1 = female; N = 374;*

* *p < 0. 05;*

*** *p < 0.001*.

### Model Testing

Before the regression analysis, we conducted the multicollinearity diagnosis on the predictor variables. The results showed that the tolerance between the predictor variables was >0.2, and the variance inflation factor (VIF) was <5. It showed that the multicollinearity between the predictor variables is not serious (Fox, 1991). The analysis results were presented in Table 2, which consists of four parts: Model 1, Model 2, Model 3, and the conditional indirect effect analysis of Model 2. Model 1 tested the effects of body image dissatisfaction on self-acceptance; Model 2 examined the effects of body image dissatisfaction, gender and self-acceptance on self-esteem; and Model 3 investigated the effects of body image dissatisfaction, self-acceptance, and self-esteem on impulse buying.

**Table 2:** Test of the moderated mediation.

	**Model 1**			**Model 2**			**Model 3**		
	**(Outcome: Self-acceptance)**			**(Outcome: Self-esteem)**			**(Outcome: impulse buying)**		
	***SE***	**β**	***t***	***SE***	**β**	***t***	***SE***	**β**	***t***
Gender				0.23	−0.62^**^	−2.71			
Age	0.01	0.02	2.68	0.01	−0.01	−1.90	0.02	0.02	1.16
Body image dissatisfaction	0.03	−0.32^**^	−9.42	0.03	−0.20^***^	−5.77	0.08	0.37^***^	4.59
Self-acceptance				0.16	0.34^*^	2.12	0.14	0.24	1.72
Self-esteem							0.12	−0.46^***^	−3.99
Self-acceptance × Gender				0.09	0.26^**^	2.87			
*R^2^*	0.21			0.58			0.16		
*F*	48.20^***^			100.36^***^			17.80^***^		

* *p < 0.05;*

** *p < 0.01;*

*** *p < 0.001*.

The conditional indirect effect analysis of Model 2 analyzed the effects of self-esteem on self-acceptance at the mean, plus and minus one standard deviation level of gender. Model 1 (*F* = 48.20, *R*^2^ = 0.21, *p* < 0.001), Model 2 (*F* = 100.36, *R*^2^ = 0.58, *p* < 0.001), and Model 3 (*F* = 17.80, *R*^2^ = 0.16, *p* < 0.001) show that body image dissatisfaction positively predicted impulse purchasing (β = 0.37, *p* < 0.001) after controlling for age. The results supported Hypothesis 1. Unexpectedly, body image dissatisfaction negatively predicted self-acceptance (β = −0.32, *p* < 0.01), but self-acceptance cannot predict impulse buying (β = 0.24, *p* > 0.05). The results didn't support Hypothesis 2. body image dissatisfaction negatively predicted self-esteem (β = −0.20, *p* < 0.001), and self-esteem negatively predicted impulse buying (β = −0.46, *p* < 0.001). Hypothesis 3 was supported. Body image dissatisfaction negatively predicted self-acceptance (β = −0.32, *p* < 0.01) and self-acceptance positively predicted self-esteem (β = 0.34, *p* < 0.05), and self-esteem negatively predicted and impulse buying (β = −0.46, *p* < 0.001). Thus, Hypothesis 4 was supported.

**Figure 2:**
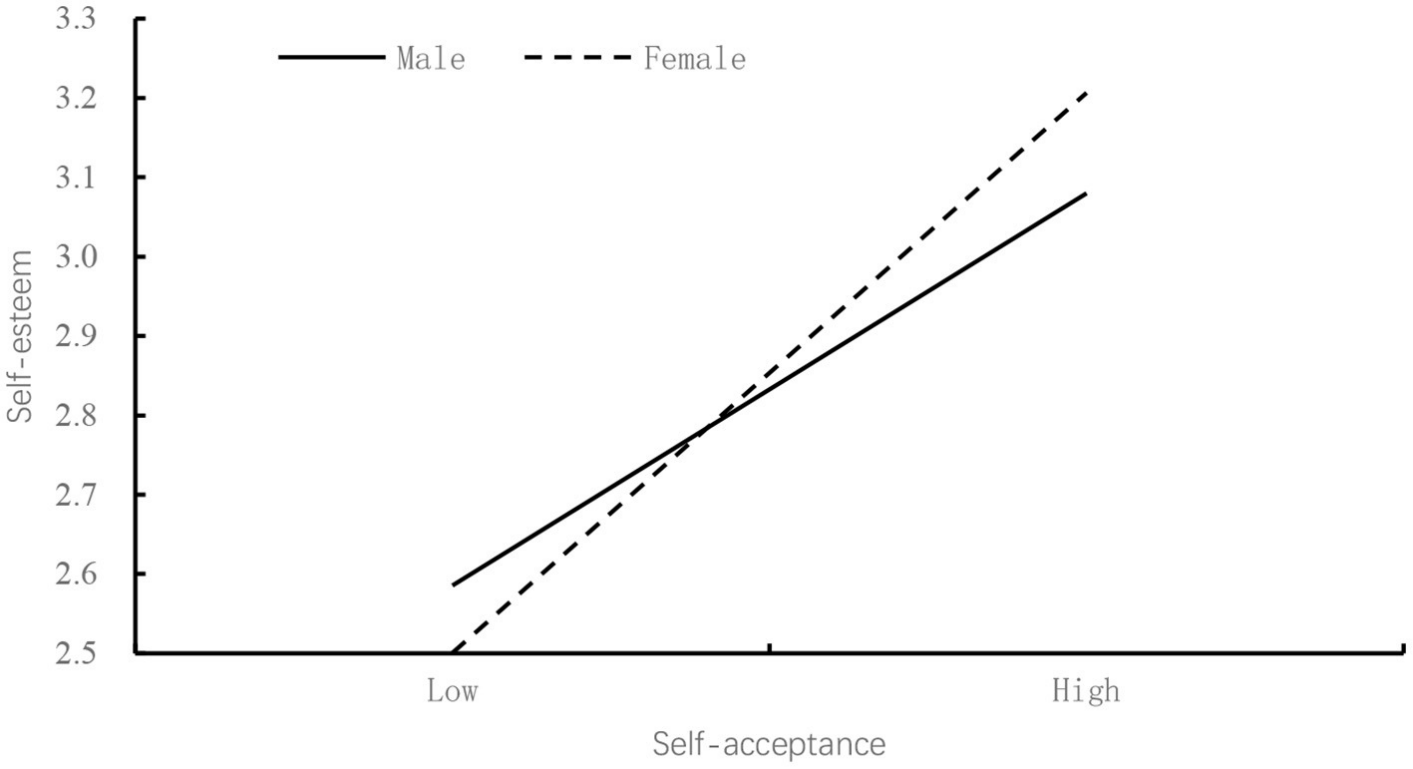
Gender moderates the relation between Self-esteem and Self-acceptance.

Our findings indicated that self-acceptance positively predicted self- esteem (β = 0.34, *p* < 0.05), and the predictive effect of self-acceptance on self-esteem was moderated by gender. In order to explain the essence of the interactive effect of gender and self- acceptance more clearly, we divided the subjects into a female group and a male group according to gender, and used a simple slope test to analyze the moderating role of gender in self-acceptance and self-esteem (Aiken and West, 1991). The results were shown in Figure 2. Simple slope tests showed that the association between self-acceptance and self-esteem was stronger for female (β_simple_ = 0.85, *p* < 0.001) than for male (β_simple_ = 0.59, *p* < 0.001). Therefore, Hypothesis 5 was supported.

## Discussions and Conclusions

This study explores the relationship of body image dissatisfaction on impulsive buying and its internal mechanism from the perspective of body image under the guidance of theories such as self-regulation and compensatory consumption. On the one hand, we elucidate how body image dissatisfaction plays a role, namely through the mediating effect of self-acceptance and self-esteem to relate to impulsive buying behavior; On the other hand, this paper analyzes when this effect is greater, that is, the intermediate path of this integrated mediation process is regulated by gender. The moderating effect is manifested in the fact that women's external self-acceptance had a greater predictive effect on self-esteem than men's. The results of this study are of great significance for further exploring the formation mechanism of impulsive buying and advocating correct and rational consumption behavior.

Previous studies mostly focused on the impact of consumers' regulatory orientation, impulsive trait and vanity characteristics on impulsive buying behavior (Sengupta and Zhou, 2007; Niu and Liu, 2015; Xie et al., 2015). This study explores the relationship between body image dissatisfaction and impulsive buying. The results show that body image dissatisfaction is positively correlated with impulsive buying, which is consistent with previous studies (Lucas and Koff, 2017). The possible explanation for the results is that impulse buying was a preventative self-regulation strategy. When negative emotions are caused by unsatisfactory evaluation of body image, individuals make impulse buying to alleviate the pain caused by perception of negative body image. Therefore, body image dissatisfaction plays a significant role in promoting impulsive buying behavior.

The hypothesis that self-acceptance mediated the relation between body image dissatisfaction and impulsive buying was not supported. That is, self-acceptance is not a possible reason that some people engage in compulsive buying in response to body image dissatisfaction. Body image dissatisfaction is closely related to self-acceptance, which is consistent with previous research evidence that body image dissatisfaction negatively predicts self-acceptance (Xie et al., 2018). But the study found that self-acceptance did not significantly predict impulse buying. This may be because self-acceptance is a self-directed attitude, and the level of self-acceptance leads to different emotional experiences. Although studies have shown that high self-acceptance is accompanied by positive emotions (Jimenez et al., 2010), and positive emotions promote impulsive buying (Fan and Zhang, 2006; Lu et al., 2015). However, some researchers have pointed out that people with low self-acceptance are often accompanied by negative emotions such as depression, self-blame and self-hatred (Sun and Lu, 2017). In other words, self-acceptance is negatively correlated with negative emotions (Chamberlain and Haaga, 2001; Cunha and Pavia, 2012). At the same time, Maxwell and Kover (2003) believe that impulsive buying is driven by negative emotions, and individuals could improve their bad mood by buying their favorite goods. In other words, it is not only high self-acceptance that may promote impulsive buying, but low self-acceptance may also lead to impulsive buying. Therefore, the relationship between self-acceptance and impulsive purchase is unclear and needs further discussion in the future.

According to our hypothesis, self-esteem plays a mediating role between body image dissatisfaction and impulsive buying. This study explored the relationship between these three variables and confirmed that body image dissatisfaction could negatively predict self-esteem and positively predict impulsive buying. One possible reason is that individuals may underrate their self-worth when they think they are not beautiful or attractive enough, and thus perceive themselves as far from the ideal image presented in the media. This low self-worth is low self-esteem. When a person with low self-esteem is exposed to a compensatory product of interest in a buying situation, he/she has an irresistible desire (the purchase impulse) to purchase a product that helps bridge the gap between the real self and the ideal self (Wicklund and Gollwitzer, 1981; Dittmar et al., 1996), in order to get rid of the psychological pressure related to the sense of self-inadequacy (Youn and Faber, 2000).

In addition, the results further found that body image dissatisfaction positively predict impulsive purchase through self-acceptance and self-esteem, which is consistent with the previous findings that self-acceptance is beneficial to improve self-esteem (Gao and Cong, 2000; Zhang, 2016; Zhu, 2016). The results show that self-acceptance and self-esteem are able to serve as a sequential mechanism that links body image satisfaction to impulsive buying tendency, such that this process begins with the self-image dissatisfaction, continues with low levels of self-acceptance and negative self-value evaluation as a psychological state and ends with negative emotions toward body. Therefore, for the public, positive evaluation of their own body image, objective view of themselves, acceptance of everything about themselves, and high self-value evaluation may all play a role in restraining impulsive buying behavior.

According to our hypothesis, gender moderates the relationship between self-acceptance and self-esteem. As explained by the social identity theory, individuals in a social environment will attribute themselves to a certain group based on their gender, identity and other information, and they will identify with this group social norms, and then construct self on the basis of identity to gain self-esteem when individuals think they belong to a certain group (Tajfel, 2003). Under the impact of social norms, people of different genders have different characteristics. Men are stressed that they should have good qualities such as ambition or sense of responsibility, instead of having a better appearance (Josephs et al., 1992). Moreover, men will internalize this social norm and construct themselves based on it. When men have problems in their abilities and qualities, they are threatened by social norms and have more negative emotions toward themselves (Heilman et al., 1995), which leads to a decrease in their self-acceptance. However, women's education and growing environment make them pay more attention to appearance than men. Women are influenced by social and cultural factors to attach more importance to their physical attractiveness. Once they ignore their attention to their appearance, they will have a lower acceptance of themselves (Wang and Guo, 2013). Therefore, when there is a problem with the self-concept that individual value more, it will cause them to have doubts about their self-worth and lead to lower self-acceptance. The emotional model of self-esteem (Brown, 1993) holds that self-esteem is a kind of self-acceptance with emotion, which reflects an individual's love and acceptance of himself. Therefore, such negative self-worth and low self-acceptance could have a negative impact on an individual's self-esteem (Berg et al., 2007). In short, there are gender differences in the importance people attach to self-concept. Due to the influence of gender norms and negative emotions, this gender difference is reflected in the predictive effect of the internal and external self on self-esteem. Specifically, men who were more accepting of positive self-traits had higher self-esteem; Women who were more accepting of their appearance had higher self-esteem.

## Limitations and Implications

There are some deficiencies in this study, which need to be further improved in future studies. First of all, cross-sectional design is adopted in this study. Although previous theoretical and empirical evidence provided a solid foundation for this study, it is difficult to infer the causal relationship between variables. In the future, researchers could combine experiments and follow-up studies to explore the mechanism of action between variables. Secondly, all the data in this study are from self-reports of the respondents. Although the common method deviation is not obvious, there may still be social approval effect. For example, college students tend to focus on and change their body image. As a result, they may give dishonest answers in order to cater to the mainstream “thin as beauty” or “muscular as beauty.” Future research could attempt to collect data from multiple sources.

Although the current research has some limitations, it still has some important significance. Impulse buying has become a common phenomenon in people's lives (Liang et al., 2008). Therefore, it is of great application value to explore the mechanism between body image dissatisfaction and impulse buying. From the perspective of body image, this study discusses the impact of body image dissatisfaction on college students' impulsive buying and its internal mechanism of action, which is conducive to guiding the public to form an objective and correct evaluation of their appearance and body, thus helping them to reduce their impulsive buying behavior and become rational consumers. At the same time, this study could also provide guidance for people to reduce the negative impact of impulsive buying on psychology and life, improve people's mental health and living conditions, and thus improve life satisfaction and subjective happiness. Teachers could carry out mental health counseling activities in a targeted manner to guide students to establish a correct outlook on the body. Various activities aimed at affirming self-worth could be organized on campus. For students, they should not be overly indulged in the figure beautified by the media, but instead, should explore their inner strengths, enhance their self-identity, and become sensible consumers. In addition, the moderated effect of gender suggests that we need to take different interventions for different genders in impulsive buying behaviors.

## Data Availability Statement

The raw data supporting the conclusions of this article will be made available by the authors, without undue reservation.

## Ethics Statement

The studies involving human participants were reviewed and approved by Ethical Committee of School of Psychology of Central China Normal University. Written informed consent for participation was not required for this study in accordance with the national legislation and the institutional requirements.

## Author Contributions

ZC led the study design, data collection, statistical analysis, and drafted the manuscript. YG helped to analyze the data and draft the manuscript. DW and HY helped to design the study and collect data. PM and ZW participated in the interpretation of the data. All authors read and approved the final manuscript.

## Conflict of Interest

The authors declare that the research was conducted in the absence of any commercial or financial relationships that could be construed as a potential conflict of interest.
